# Cloning and Functional Characterization of a Pericarp Abundant Expression Promoter (*AhGLP17-1P*) From Peanut (*Arachis hypogaea* L.)

**DOI:** 10.3389/fgene.2021.821281

**Published:** 2022-01-20

**Authors:** Yasir Sharif, Hua Chen, Ye Deng, Niaz Ali, Shahid Ali Khan, Chong Zhang, Wenping Xie, Kun Chen, Tiecheng Cai, Qiang Yang, Yuhui Zhuang, Ali Raza, Weijian Zhuang

**Affiliations:** ^1^ College of Plant Protection, Fujian Agriculture and Forestry University (FAFU), Fuzhou, China; ^2^ Fujian Provincial Key Laboratory of Plant Molecular and Cell Biology, Oil Crops Research Institute, Fujian Agriculture and Forestry University (FAFU), Fuzhou, China; ^3^ Center of Legume Crop Genetics and Systems Biology, College of Agriculture, Fujian Agriculture and Forestry University (FAFU), Fuzhou, China

**Keywords:** cis-elements, GUS staining, pathogens, tissue-specific expression, transgenic arabidopsis

## Abstract

Peanut (*Arachis hypogaea* L.) is an important oil and food legume crop grown in tropical and subtropical areas of the world. As a geocarpic crop, it is affected by many soil-borne diseases and pathogens. The pericarp, an inedible part of the seed, acts as the first layer of defense against biotic and abiotic stresses. Pericarp promoters could drive the defense-related genes specific expression in pericarp for the defense application. Here, we identified a pericarp-abundant promoter (*AhGLP17-1P*) through microarray and transcriptome analysis. Besides the core promoter elements, several other important *cis*-elements were identified using online promoter analysis tools. Semiquantitative and qRT-PCR analyses validated that the *AhGLP17-1* gene was specifically expressed only in the pericarp, and no expression was detected in leaves, stem, roots, flowers, gynophore/peg, testa, and embryo in peanut. Transgenic *Arabidopsis* plants showed strong GUS expression in siliques, while GUS staining was almost absent in remaining tissues, including roots, seedlings, leaf, stem, flowers, cotyledons, embryo, and seed coat confirmed its peanut expressions. Quantitative expression of the *GUS* gene also supported the GUS staining results. The results strongly suggest that this promoter can drive foreign genes’ expression in a pericarp-abundant manner. This is the first study on the functional characterization of the pericarp-abundant promoters in peanut. The results could provide practical significance to improve the resistance of peanut, and other crops for seed protection uses.

## Introduction

Peanut (*Arachis hypogaea* L.), also known as groundnut, is an important legume crop (Fabaceae family) that is mainly cultivated for its edible seeds. Botanically peanut is a legume, but it is frequently eaten as a “nut”, and its nutritional value is comparable to other nuts (Settaluri et al., 2012). It is a major nutrition source for humanity, providing edible oil and proteins (Khan et al., 2020) after soybean and rapeseeds. Peanut is full of nutrients, including carbohydrates, vitamins, lipids, minerals, etc. (Akhtar et al., 2014). Peanut is widely cultivated globally in tropical and subtropical regions, producing 48.75 million tons of shells annually. China is the leading producer with 17.52 million tons of shells and shares 36% of the Worlds’ total production, followed by India (14%), Nigeria, Sudan, and the USA (FAOSTAT, 2019).

Like other crops, the peanut is affected by several bacterial, fungal, and viral pathogens (Ali et al., 2020). To manage these pathogens, nature has provided peanuts with several defense mechanisms. As peanut seeds are of primary importance, they have evolved to form non-edible outer coverings (pericarp and testa) to protect the seeds from insects, pathogens, and physical damage and maintain seed viability from generation to generation (Souza and Marcos-Filho, 2001). The shape, color, thickness, etc., of the seed coat, may differ among species during the evolutionary process and due to different growing environments. Biologically active chemicals in seed coats provide the right solution for infections, especially seed coat-based pathogen resistance attributed to hydrophobic molecules like lignin and tannins (Dardick and Callahan, 2014). Seed inner pericarp tissues also accumulate different antifungal and antibacterial metabolic by-products and flavonoids (Dixon and Paiva, 1995). Plenty of work is available on the genetic basis of seed coat development in the model dicot plant, i.e., *Arabidopsis*, and many genes have been worked out for their functions in seed coat development (Windsor et al., 2000; Haughn and Chaudhury, 2005; Dardick and Callahan, 2014). Similarly, detailed knowledge about the genes involved in seed coat development in peanut can help manipulate specific genes to get resistance against specific biotic or abiotic stresses. Paik-Ro and his team have reported that the cDNA of *PSC33I* is specifically expressed in peanut seed tissues without showing any expression in other tissues (Paik-Ro et al., 2002).

The physical appearance of any plant and its response to the surrounding environment is mainly controlled by a cascade of genes. Expression of any gene depends upon the binding of specific transcription factors (TFs) or proteins with unique upstream elements (*cis*-elements) and regulation at the transcription site. Promoters are the non-coding regulatory DNA sequences present upstream of a genes’ coding region and contain specific *cis*-elements that regulate the spatio-temporal expression of a gene. A promoter has multiple binding sequences for TFs and RNA polymerase for activation and regulation of functions of the downstream gene (Ong and Corces, 2011; Gupta et al., 2012; Hernandez-Garcia and Finer, 2014). Promoters are important tools for molecular research to study the functions of a gene (Xu et al., 2016). Promoters can be divided into constitutive, spatio-temporal/tissue-specific, inducible, and synthetic promoters based on regulating the gene functions. Constitutive promoters drive the expression of a gene in all tissues, such as maize ubiquitin promoter (Christensen et al., 1992) and cauliflower mosaic virus promoter (CaMV35S) (Odell et al., 1985), are widely used for functional gene studies in plants.

Similarly, Figwort mosaic virus sub-genomic transcript promoter *FMV Sgt* is another example of constitutive promoters (Bhattacharyya et al., 2002). The constitutive expression of an exogenous gene can cause adverse effects on a plants’ normal growth and functioning as it causes an extra burden on plant metabolism for expressing a gene in tissues where it is not required, and sometimes it results in undesired phenotypes (Nakashima et al., 2007). Tissue-specific and inducible promoters are more important as they can drive a genes’ expression in a tissue-specific manner or under specific stress; hence, avoiding the adverse effects of constitutive expression. The selection of a suitable promoter is the key step for developing transgenic plants since it influences the cell, tissue, or stage specificity and determines the expression level of the transgene (Koyama et al., 2005). Therefore, comprehensive knowledge of promoter activity is a prerequisite for transgenic breeding.

In this paper, we studied the promoter of a peanut pericarp abundant expression gene belonging to the Germin-like protein (GLPs) family. Germin-like proteins were first identified in germinating wheat seedlings as germination specific markers during the 1980s (Lane et al., 1993). These proteins belong to a group of water-soluble glycoproteins with diverse functions. GLPs are ubiquitously found in gymnosperms, and angiosperms (Lu et al., 2010) and are broadly involved in plants’ defense against biotic and abiotic stresses (Godfrey et al., 2007). Germin-like proteins are a group of “Cupin superfamily” containing the Cupin domain (PF00190). Structurally these proteins are composed of the β-sheet barrel (jellyroll beta-barrel structure), and the protein’s C-terminus contains a metal ion binding site (Agarwal et al., 2009). GLPs contain two conserved motifs known as “germin-box”. These motifs G(x)5HxH(x)3,4E(x)6G and G(x)5PxG(x)2H(x)3N are packed in a classic jellyroll beta-barrel structural domain (Yamahara et al., 1999). Thus far, there is no report available which investigated its specific expression and characterized its upstream promoter in a plant pericarp.

Here, this study identified and characterized a peanut *GLP* gene, “Germin-like protein subfamily 1 member 7 (*AhGLP17-1*)," showing abundant expression in seed pericarp. This gene was selected based on the transcriptome and microarray expression data, and its pericarp abundant expression was verified by semiquantitative and qRT-PCR. Further, the *cis*-regulatory elements of the promoter were analyzed, and their expression was characterized in transgenic *Arabidopsis* plants.

## Materials and Methods

### Plant Materials and Growth Conditions

Seeds of peanut cultivars Minhua-6 (M-6), and Xinhuixiaoli (XHXL), and *Arabidopsis thaliana* ecotype Columbia-0 (Col-0) were obtained from the Institute of Oil Crops, Fujian Agriculture and Forestry University, Fuzhou, China. Peanut plants (M-6) were grown in research fields at Yangzhong, Sanming county of Fujian province during the summer season (April-August, 2018), and samples from different tissues, including leaves, stem, flower, peg, pericarp, testa, embryo, and roots were collected at different growing stages for semiquantitative and qRT-PCR-based confirmation of *AhGLP17-1* gene. For isolation of promoter, peanut plants (XHXL) were grown in the greenhouse in small pots (10 cm diameter) filled with compost, and leaf samples were collected from one-week-old plants. Arabidopsis plants were germinated on MS medium (Murashige and Skoog, 1962), transferred into 8-cm diameter plastic pots containing compost after 2 weeks, and further grown in the greenhouse, where 25°C temperature and 16/8 h photoperiod for day/night cycle was maintained for *Arabidopsis* seedlings. All samples were washed with 75% ethanol and sterilized water, packed in previously labeled plastic bags, and immediately frozen in liquid nitrogen and stored at −80°C for further use.

### Selection and Verification of Pericarp-Abundant *AhGLP17-1* Gene

A pericarp abundant gene with Peanut Genome Resource (PGR) ID AH06G08990.1 was identified by the microarray and transcriptome expression data, which is available at the PGR database http://peanutgr.fafu.edu.cn/ (accessed on 20^th^ April 2018) (Zhuang et al., 2019). This gene belongs to the germin-like protein family viz. “germin-like protein subfamily 1 member 7”. Due to the polyploid nature of the peanut genome, peanut contains nine different copies of germin-like protein subfamily 1 member 7, and we named this gene *AhGLP17-1*. The protein 3D structure of *AhGLP17-1* was constructed using 3D Ligand Site https://www.wass-michaelislab.org/3dlig/index.html (accessed on 15th June 2018) (Wass et al., 2010), physio-chemical properties were predicted by Expasy server https://web.expasy.org/protparam/ (accessed on 20th June 2018) (Gasteiger et al., 2005), and subcellular localization was predicted by CELLO V2.5 http://cello.life.nctu.edu.tw/ (accessed on 22nd June 2018) (Yu et al., 2006). Gene functional annotation “gene ontology” information was retrieved from the PGR database (http://peanutgr.fafu.edu.cn/). Semiquantitative and qRT-PCR analysis were used to confirm its expression in different tissues.

### Selection of Promoter Region and Identification of *Cis*-regulatory Elements

A 2296 bp region upstream of the start codon of the *AhGLP17-1* gene was selected for promoter analysis. The promoter was named *AhGLP17-1P*. *Cis-*regulatory elements of promoter region were predicted by PLACE https://www.dna.affrc.go.jp/PLACE/?action=newplace (accessed on 30th June 2018) (Higo et al., 1998) and PlantCARE databases http://bioinformatics.psb.ugent.be/webtools/plantcare/html/ (accessed on 30th June 2018) (Rombauts et al., 1999).

### RNA Extraction, cDNA Synthesis, Semiquantitative, and qRT-PCR Analysis

RNA from different tissues (leaves, stem, flowers, peg, pericarp, testa, embryo, and roots) of peanut cultivar Minhua-6 was extracted by the Cetyl Trimethyl Ammonium Bromide (CTAB) (200 mM Tris HCL, 20 mM EDTA, 2% CTAB, 1.4 M NaCl, and 2% PVP-40, pH = 8.0, 0.2% β-Mercapto Ethanol added before use) method with some modification (Chen et al., 2016). First-strand cDNA was synthesized using 1 µg RNA with the PrimeScript 1st strand cDNA Synthesis Kit (Cat# 6110A) (Takara, Dalian, China) according to manufacturer guidelines. Semiquantitative PCR analysis was performed to check the expression of the *AhGLP17-1* gene in different tissues using the peanut *Actin* gene and gene-specific primers. The semiquantitative PCR results were viewed on 1% agarose gel. For qRT-PCR analysis, three different cDNA preparations for each tissue were used. Quantitative real-time polymerase chain reaction (qRT-PCR) was performed by MonAmp™ ChemoHS qPCR Mix (Cat# 160431) (Monad Biotech, Wuhan, China). The PCR reaction mixture contained 10 µL MonAmp Master Mix, 1 µL forward and reverse primer (diluted at 10 mM concentration), and 1 µL cDNA template. The peanut *Actin* gene (Chi et al., 2012) was used as an internal control to normalize the cDNA. The qRT-PCR reaction was performed using Applied Biosystems 7,500 real-time PCR system (ThermoFisher Scientific, USA). The cycling program was as follows: 94°C (1 min), 60°C (1 min), and 72°C (1 min) for 40 cycles. Primers used for semiquantitative and qRT-PCR analysis are given in (Supplementary Table S1).

### DNA Extraction and Isolation of Promoter

Genomic DNA from peanut (XHXL variety) leaves was extracted using the CTAB (1 M Tris-HCl, 0.5 M Na-EDTA, 2% CTAB, 1.4 M NaCl, 3% PVP-40, and 0.2% β-Mercapto Ethanol added before use) method (Li et al., 2013) with some modifications. Promoter-specific primers (Supplementary Table S1) covering 2,296 bp upstream region before start codon were used to clone *AhGLP17-1P*. The promoter region was amplified with PrimeSTAR^®^ Max DNA polymerase (Cat# Ro45B) (Takara, Dalian, China), according to manufacturers’ guidelines. The PCR amplified product was visualized on 2% agarose gel and purified by TIANGEN Universal DNA Purification Kit (Cat# DP103-03) (Tiangen Biotech Beijing, China). Purified PCR products were sub-cloned into pMD19T vector (Cat# 3271) (Takara, Dalian, China) and sequenced by Beijing Genomics Institute (BGI, Shenzhen, China).

### Vector Construction

The sequence-verified PCR clones were used to construct the expression vector using Two-step Gateway cloning. First, the promoter was amplified by primers containing universal overlapping sequences for gateway vectors (Supplementary Table S1) using the TA-cloning product as template and purified through TIANGEN Universal DNA Purification Kit (Cat# DP214-03) (Tiangen Biotech Beijing, China). The purified PCR products were ligated by Gateway BP reaction using BP Clonase enzyme (Cat# 11789020) (Invitrogen, ThermoFisher Scientific USA) into entry vector pDONR-207 between attP sites. Vector pDONR-207 containing *AhGLP17-1P* was transferred to *E. coli* (DH5α), and positive clones were selected for sequence confirmation. After sequence confirmation, *AhGLP17-1P* was ligated into destination vector pMDC164 by Gateway LR reaction using LR Clonase enzyme (Cat# 11791020) (Invitrogen, ThermoFisher Scientific USA). Expression vector pMDC164 contains the hygromycin resistance gene for positive plants’ selection and the *GUS* reporter gene. The vector was named pMDC164-*AhGLP17-1P*.

### Transformation Into Arabidopsis

Expression vector pMDC164-*AhGLP17-1P* was transferred into *Agrobacterium tumefaciens* strain GV3101, and positive colonies were selected on yeast extract beef (YEB) selection medium plates supplemented with 50 μg ml^−1^ kanamycin. Positive *A. tumefaciens* cells harboring the expression vector were grown overnight at 28°C, 220 rpm to get logarithmic growth phase (OD600 = 1.0-1.5) in liquid YEB medium supplemented with kanamycin 50 μg ml^−1^, and 75 μg ml^−1^ rifampicin. Bacterial cells were harvested by centrifugation at 4,000 rpm for 10 min and resuspended in 5% sucrose solution containing 0.02% Silwet L-77 and 100 μg ml^−1^ acetosyringone was also added to achieve higher transformation efficiency (Sheikholeslam and Weeks, 1987). Mature *Arabidopsis* plants were used for transformation by floral dip method (Clough and Bent, 1998) by dipping the unopened flowers into the prepared solution for 10–15 s, and then placed under dark for 24 h. Siliques and opened flowers were removed before the transformation, and the floral dipping was repeated after 5 days. After that, plants were grown under optimum growth conditions until seeds were ready to harvest, and finally, the T0 seeds were harvested.

### Screening of Positive Transgenic Plants

To identify positively transformed plants, T0 transgenic seeds were screened on MS medium containing 50 μg ml^−1^ hygromycin. First, seeds were surface sterilized with 75% ethanol for 2 min and then treated with 10% H_2_O_2_ for 2 min, followed by 4–5 times washing with distilled water. Then seeds were spread over MS medium containing plates supplemented with Hygromycin antibiotic. Eight randomly selected hygromycin-resistant plants were verified by PCR amplification with promoter-specific forward and *GUS* gene specific reverse primers (Supplementary Table S1). The selected transgenic plants were covered with plastic sheets to avoid the chances of cross-pollination, and T1 transgenic seeds were obtained. Transgenic seeds were grown to further generations to get homozygous T3 lines.

### Histochemical GUS Staining and Expression Analysis of *GUS* Gene

For the GUS staining assay (Jefferson et al., 1987), tissue samples were incubated in 2 mM 5-Bromo-4-chloro-3-indolyl β-D-glucuronide (X-Gluc) solution prepared in 0.1% Triton X-100, 50 mM sodium phosphate buffer, 10 mM EDTA, 2 mM potassium ferricyanide, and 2 mM potassium ferrocyanide. Plant samples were incubated in the above-prepared staining solution at 37°C for 12 h. After staining, samples were washed with 50, 75, and 100% ethanol for 5 min separately. Finally, samples were decolorized by incubating in 75% ethanol at 37°C until all green color was removed, while ethanol was changed every 4 hours. Samples were photographed with the digital camera and Olympus microscope Model BX3-CBH with attached Olympus DP80 digital camera (Olympus, Tokyo, Japan). Quantitative expression of the GUS gene in different tissues of transgenic *Arabidopsis* plants was analyzed by qRT-PCR with *GUS* gene-specific primers (Supplementary Table S1).

For pericarp sampling, young siliques were carefully opened by sharp needles and forceps, and seeds were removed. Siliques without seeds were stored for RNA extraction. Similarly, seeds were used to examine testa, cotyledon, and embryo expression. It was impossible to separate the testa from cotyledons and get enough samples for RNA extraction from Arabidopsis seeds, so whole seeds were used to extract the RNA. RNA from different tissues of transgenic Arabidopsis was extracted using TriQuick Reagent (Cat# R1100) (Solarbio, Beijing, China), following the manufacturers’ instructions.

### Cryostat Sectioning of Transgenic *Arabidopsis* Seeds

The seeds of transgenic Arabidopsis plants were ruptured and incubated in the GUS staining solution to confirm whether GUS staining is present in testa, cotyledons, and embryo or not. After overnight incubation in GUS solution, seeds were further processed for cryostat sectioning in Leica CM1950 Cryostat Microtome (Leica Biosystems, Germany). The cryostat microtome was turned on for 5 hours before use, and the temperature was set at −20°C. Specimen discs, brushes, and forceps were put inside the cooling chamber. The freezing compound was applied on specimen discs, and seeds were gently placed on specimen discs containing the freezing compound. Specimen discs containing the seeds were kept at −20°C for 30 min. After that, 50 µm sections were made and placed on glass slides. Later, images of sectioned specimens were taken by Olympus IX73 microscope with attached Olympus DP80 digital camera (Olympus, Tokyo, Japan).

## Results

### Selection and Characterization of Pericarp Abundant Gene

**FIGURE 1:**
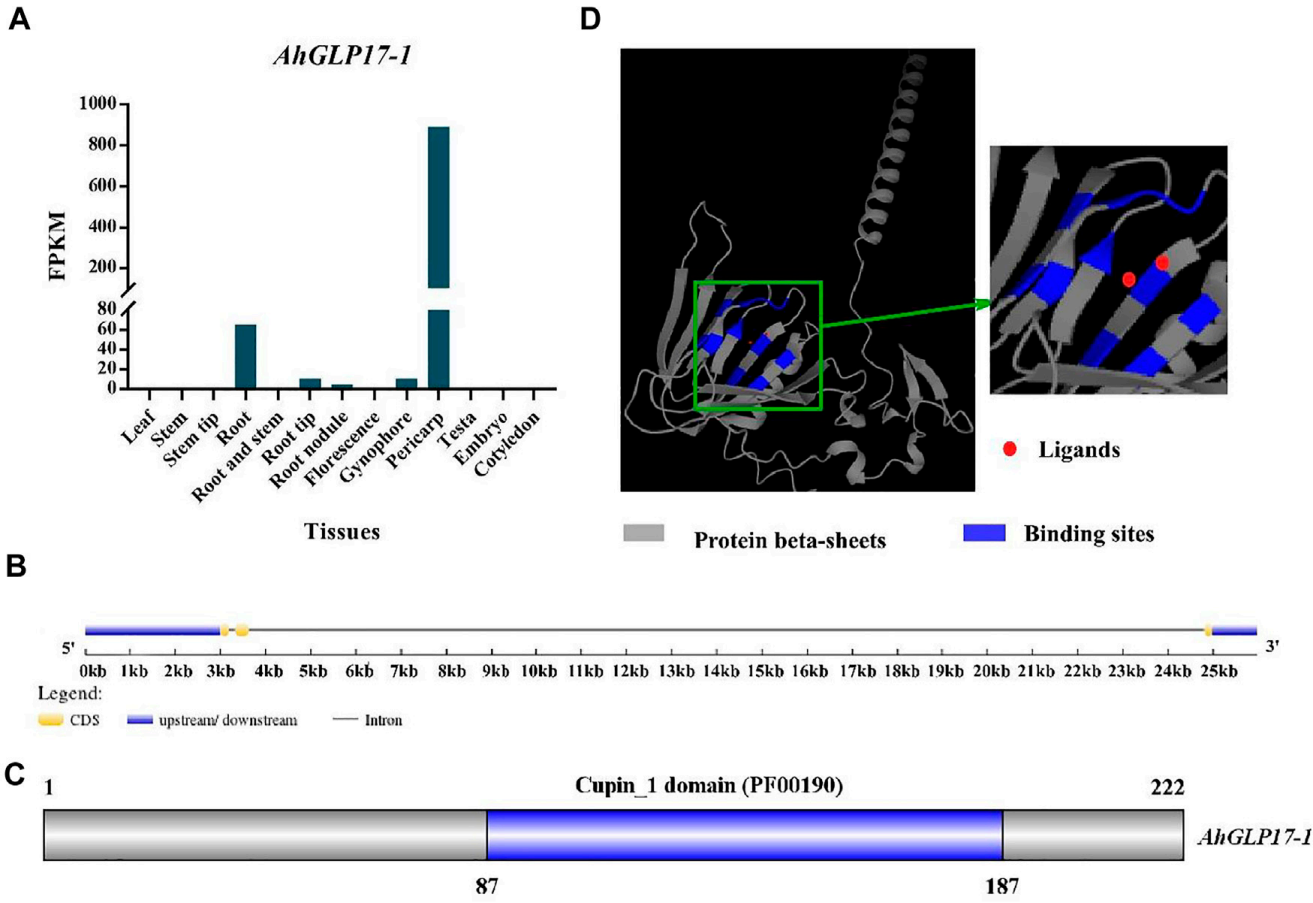
Expression and characterization of *AhGLP17-1* gene. **(A)** Transcriptome expression (FPKM values) of the *AhGLP17-1* gene in different tissues of peanut (average values of the pericarp, testa, and embryo transcriptome expression are used). **(B)** Gene structure of *AhGLP17-1*. **(C)** The position of cupin_1 domain. **(D)**The 3D protein structure of *AhGLP17-1*. Where grey color shows the protein β-sheets, blue color shows binding sites, and red dots show ligands.

We searched the Peanut Genome Resource database (http://peanutgr.fafu.edu.cn/) (Zhuang et al., 2019) for the candidate gene with high transcriptome and microarray expression in pericarp and with no or very low expression in other tissues for cloning of promoter. A member of the peanut germin-like protein family (*AhGLP*) named germin-like protein subfamily 1 member 7 (*AhGLP17-1*) with the PGR gene ID AH06G08990 and mRNA ID AH06G08990.1 was found to be specifically showing high expression in pericarp as compared to other tissues (Figure 1A). Although microarray expression was found in root tissues and gynophore/peg (Supplementary Table S2), some transcriptome expression was also present in roots and peg (Supplementary Table S3). Still, the tendency of expression of the *AhGLP17-1* gene was higher in the pericarp (Figure 1A). This gene is present on the 6th chromosome of sub-genome A at 12182490-12204462 position on the negative strand and has a CDS length of 666 base pairs and 21973 base pairs genomic length. The protein, CDS, and promoter sequences of the *AhGLP17-1* gene are given in Supplementary file S1. It consists of three exons of almost the same size and two introns, one of which is 21170 bp and the second intron is 134 bp long (Figure 1B). *In silico* subcellular localization showed that *AhGLP17-1* is localized in extracellular spaces and plasma membrane. Protein comprises 222 amino acid residues with a molecular weight of 24.49 KDa and a theoretical isoelectric point of 9.36. Further, it contains the Cupin_1 domain (PF00190) at 87-187 aa position (Figure 1C). The protein 3D structure prediction showed that *AhGLP17-1* is composed of the β-sheet barrel (jellyroll beta-barrel structure) with the ligands (Figure 1D). GO functional annotation revealed that the *AhGLP17-1* gene participates in three categories, including biological process in oxalate metabolic process (GO:0033609), molecular functions in nutrient reservoir activity (GO:0045735), oxalate decarboxylase activity (GO:0046564), manganese ion binding (GO:0030145), and cellular components apoplast (GO:0048046), cell wall (GO:0005618), and extracellular region (GO:0005576) (Supplementary Table S4). Other related information (orthologues in other plant species) is given in Supplementary Table S5.

### Validation of Pericarp Abundant Expression by Semiquantitative and qRT-PCR

**FIGURE 2:**
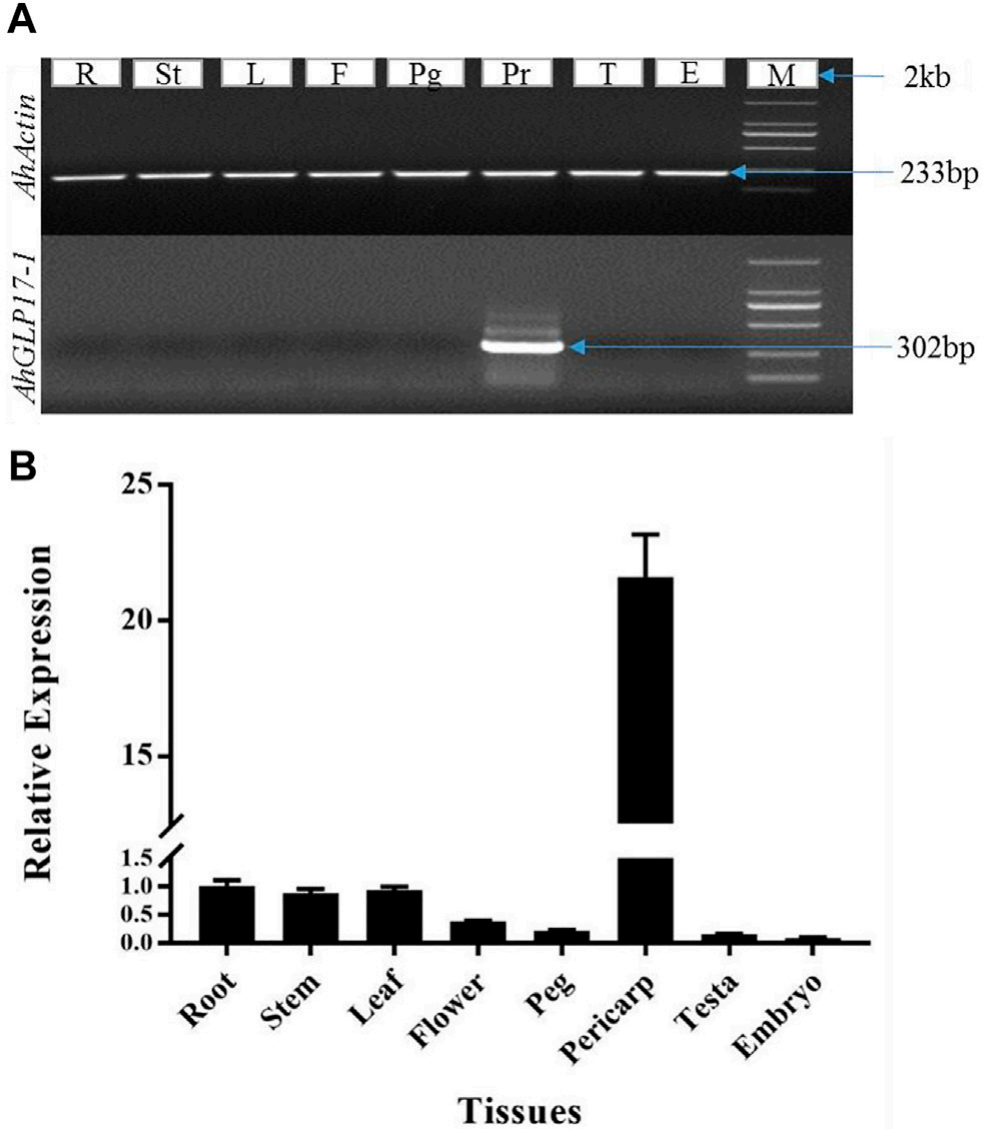
Expression analysis of *AhGLP17-1* gene expression in different tissues. **(A)** Semiquantitaive PCR-based expression analysis, **(B)** qRT-PCR-based expression analysis of *AhGLP17-1* gene expression. Both semiquantitative and qRT-PCR results are consistent with transcriptome and microarray expression data (as shown in Figure 1A). L = leaf, St = stem, Fl = flower, Em = embryo, Ts = testa, peg = peg/gynophore, peri = pericarp, and R = root. Root expression was used as a control to analyze the data.

Pericarp-abundant expression of *AhGLP17-1* among different tissues was confirmed by semiquantitative PCR and qRT-PCR. The peanut *Actin* gene was used as an internal control for both semiquantitative and qRT-PCR analysis. The peanut actin gene showed a bright band in RNA samples of all tissues, while the *AhGLP17-1* gene showed a bright band in the pericarp samples, but no expression was detected in all other tissues (Figure 2A). Results of semiquantitative PCR showed that *AhGLP17-1* was preferentially expressed in the pericarp. On the other hand, the qRT-PCR results clearly showed that a high level of transcripts of *AhGLP17-1* was present in the pericarp with minute expression in all other tissues (Figure 2B). Both semiquantitative PCR and qRT-PCR results showed that the *AhGLP17-1* gene was specifically expressed in the pericarp. It showed very minute expression in all other tissues.

### Analysis of *Cis*-regulatory Elements of *AhGLP17-1* Promoter

**FIGURE 3:**
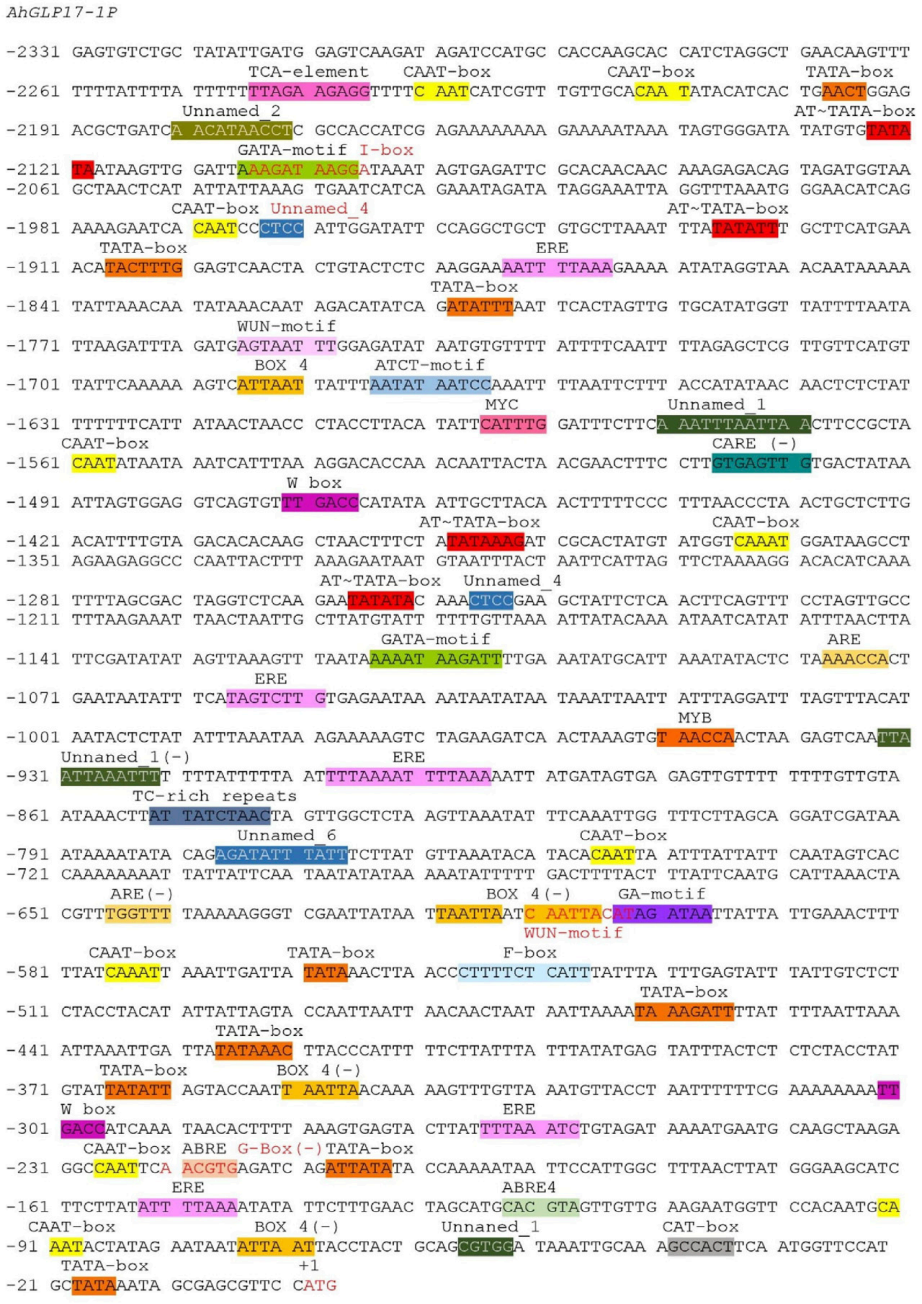
Sequence analysis of *AhGLP17-1P* promoter. Presence of *cis*-elements in promoter sequences predicted by the PlantCARE database.

A 2296 bp upstream sequence of *AhGLP17P-1* contained the basic promoter elements, including the TATA box, the key element for precise transcription initiation (Grace et al., 2004), and the CAAT box required for tissue-specific activity (Shirsat et al., 1989). Many other important regulatory elements, including light-responsive elements (ATCT-motif, Box 4, G-Box, GA-motif, GATA-motif, GT1-motif, and Gap-box); hormones-responsive elements including salicylic acid (TCA-element), gibberellin (TATC-box), ethylene (ERE), and abscisic acid (ABRE) were also predicted in the *AhGLP17-1P*. Moreover, defense-related elements (TC-rich repeats and MYB binding sites), wound responsive element (WUN-motif), and anaerobic induction responsive element (ARE) were also found inside the promoter region. Further information on *cis*-regulatory elements and their position in *AhGLP17-1P* is presented in Figure 3.

Analysis of *cis*-elements by the PLACE database revealed the presence of a number of important elements, including seed-specific elements (RY-element) and transcription factor binding sites. The details of transcription factor binding sites and other elements are given in Supplementary Table S6. The presence of these regulatory elements strongly suggests that this promoter can be suitable substitute for a genes’ native promoter. Except for these already reported *cis*-elements, some unknown elements were also found in the promoter region of the *AhGLP17-1* gene (Figure 3).

### Cloning of Promoter, Vector Construction, and Transformation

**FIGURE 4:**
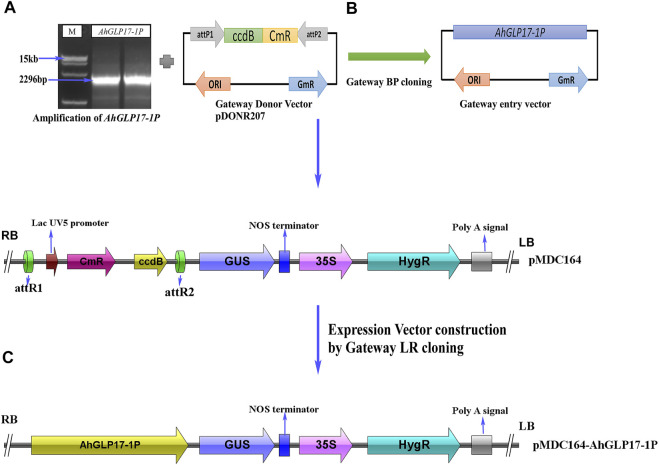
Construction of vectors using the backbone of pMDC164 vector by Gateway cloning. **(A)** Amplification of *AhGLP17-1P* promoter, **(B)** construction of Gateway entry vector by Gateway BP-cloning, **(C)** construction of Gateway expression vector using the backbone of binary vector pMDC164.

A 2296bp region for *AhGLP17-1P* was PCR amplified (Figure 4A) from the DNA template of a high yielding and fungal pathogens resistant peanut variety XHXL (Khan et al., 2020) by promoter-specific primers (Supplementary Table S1). After confirmation of sequence, the amplified promoter fragment was again amplified with gateway primers containing gateway adapter sequences and then ligated into the *attP* sites of entry vector pDONR207 by Gateway BP-cloning reaction (Figure 4B). The sequence was confirmed again after BP-cloning, and the promoter fragment was ligated into the *attR* sites of expression vector pMDC164 by Gateway LR-cloning reaction. The resulting expression vector *pMDC164-AhGLP17-1P* (Figure 4C) was transformed to *Agrobacterium tumefaciens* competent cells by heat shock method. Positive Agrobacterium colonies harboring the expression vector were selected on selection medium (YEB plates containing kanamycin and rifampicin antibiotics). Positive agrobacterium colonies were used to transform the *Arabidopsis* plants through the floral dip method, and hygromycin resistant T0 transgenic plants were screened on MS plates containing 50 μg ml^−1^ hygromycin antibiotic. Non-transformed plants turned yellow on hygromycin selection medium, while positively transformed plants were dark green and healthy, and these plants were transplanted into plastic pots containing compost. These hygromycin-resistant plants were also verified by PCR amplification.

### Characterization of the Promoter in Transgenic Plants

**FIGURE 5:**
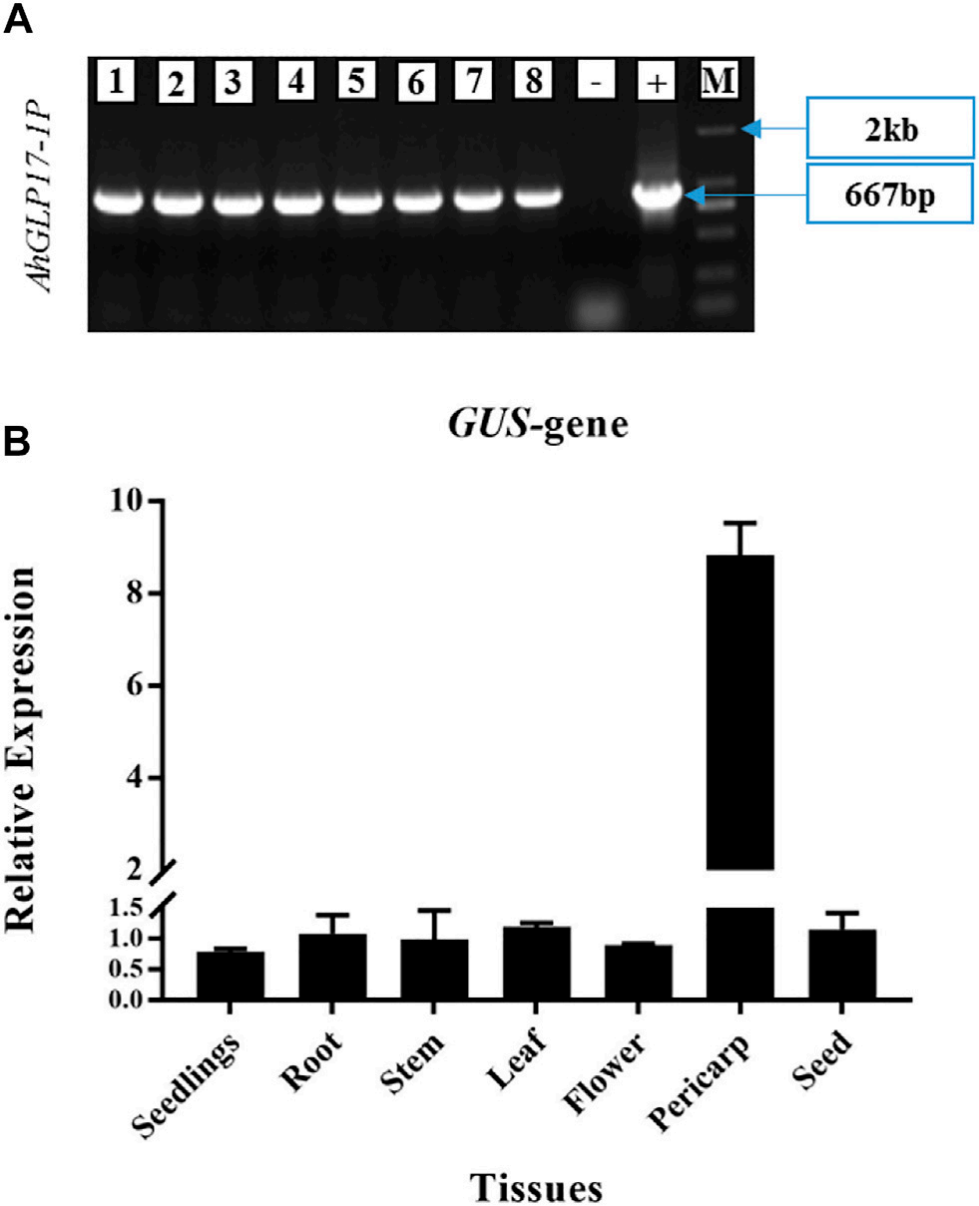
**(A)** Confirmation of T0 transgenic *Arabidopsis* plants transformed with *AhGLP17-1P* (667 bp fragment). Eight hygromycin-resistant plants verified by PCR amplification with promoter-specific forward and *GUS* gene specific reverse primer. *Arabidopsis* Col-0 was used as –ve control, and Gateway LR constructs were used as + ve control for PCR verification. M shows 2 kb marker, **(B)** Quantitative expression of *GUS* gene driven by *AhGLP17-1P* in different tissues of transgenic *Arabidopsis* plants. Root expression was used as control to analyse the data.

Hygromycin resistant positive transgenic plants were confirmed by PCR amplification using DNA as the template with promoter-specific forward and *GUS* gene specific reverse primers. While *Arabidopsis* Col-0 plants were used as a negative control, and Gateway LR constructs were used as a positive control for PCR confirmation. Eight hygromycin-resistant plants were confirmed by PCR amplification (Figure 5A). Seeds of eight positively transformed plants were sown to get the T1 generation. In T1 generation again, eight plants were selected based on hygromycin resistance and PCR confirmation. Eight selected plants of the T1 generation were covered to avoid cross-pollination, and in this way, homozygous T3 generation was obtained. Histochemical GUS expression was checked in different tissues at different growth stages in the T3 generation.

**FIGURE 6:**
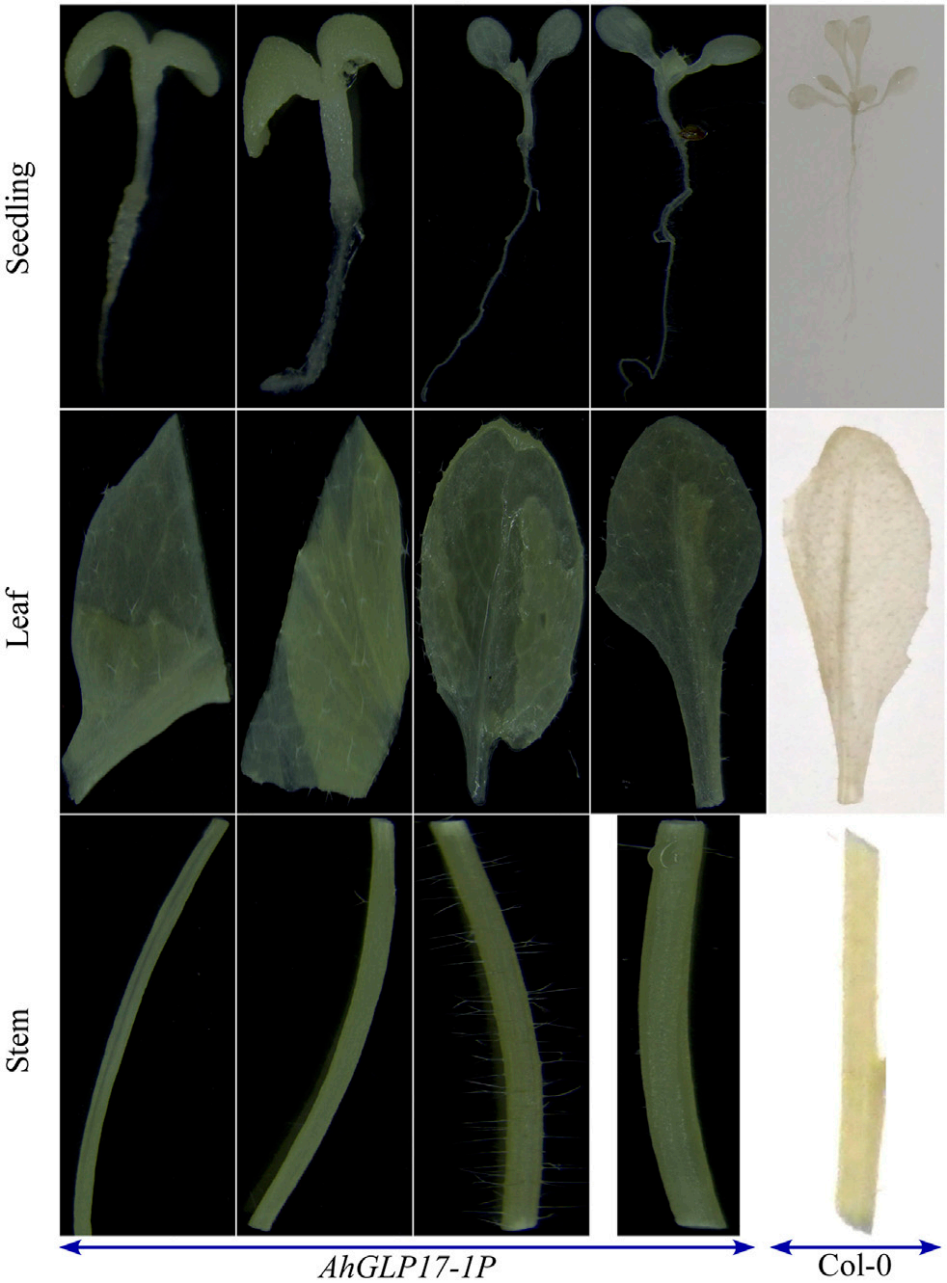
GUS staining of different vegetative tissues of *Arabidopsis* transgenic plants. *AhGLP17-1P* plants showed no staining in any vegetative tissue (seedlings, roots, leaf, and stem). Different vegetative tissues of wild-type (Col-0) plants were also used for GUS staining to compare the results.

**FIGURE 7:**
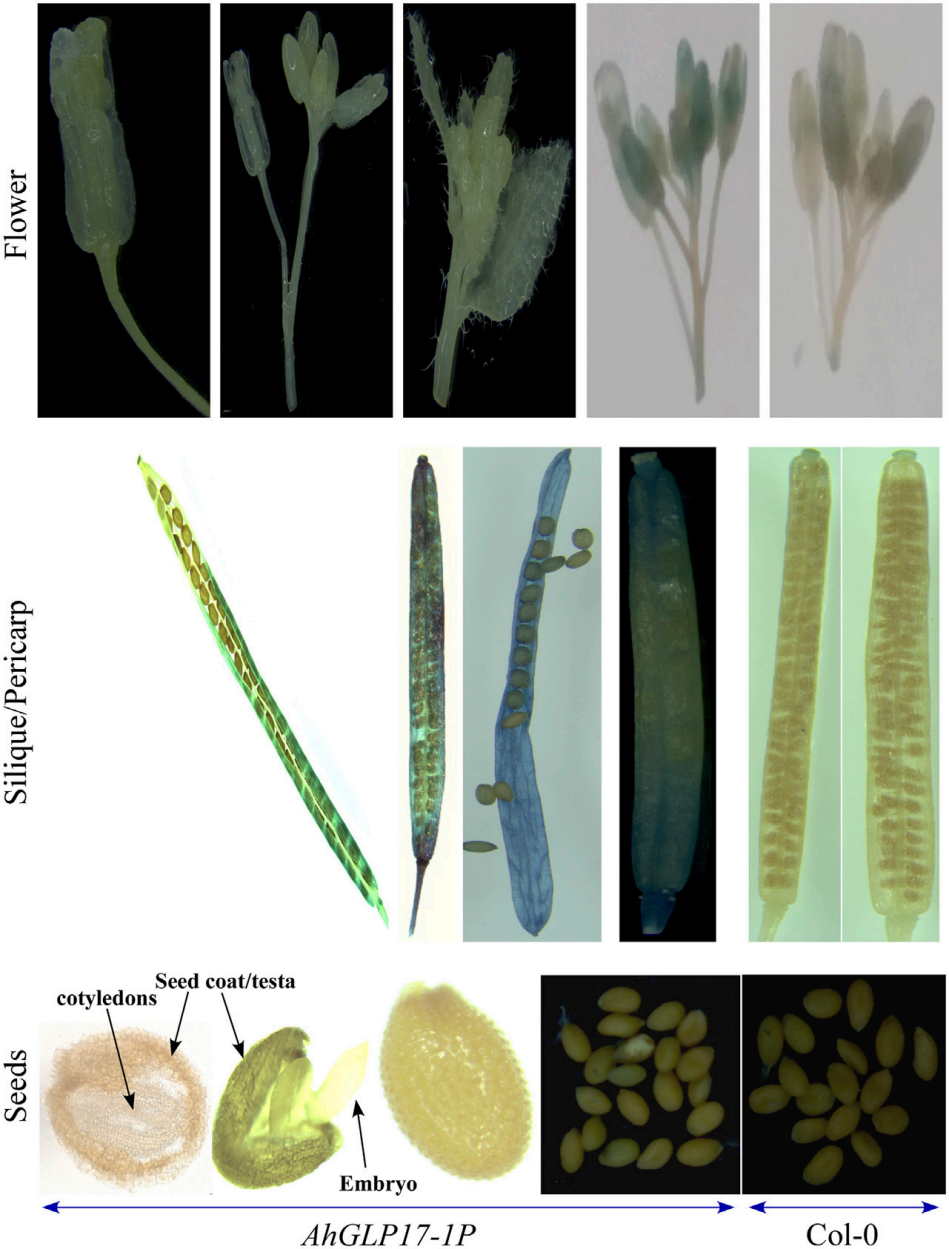
GUS staining of different reproductive tissues/organs of transgenic *Arabidopsis* plants. *AhGLP17-1P* plants showed no staining in flowers (a minute staining in some cases). Seed outer covering (pericarp) showed good staining. While staining was not present in any seed tissue (testa, cotyledons, and embryo).

The quantitative expression of the *GUS* gene was analyzed in different tissues of transgenic plants by qRT-PCR (Figure 5B). qRT-PCR results showed a relatively higher transcript level of the *GUS* gene in the pericarp of transgenic plants and a very low transcript level in other tissues (Figure 5B). GUS staining was not detected in all vegetative tissues and young seedlings (Figure 6). Among reproductive organs, a moderate level of GUS staining was present in siliques, and mild staining was also present in flowers in some cases; staining was not present in seeds. To confirm that GUS staining is not present in seed coat/testa, cotyledons, and embryo, the ruptured seeds incubated in GUS staining solution were further processed for cryostat sectioning. Staining was not found in any of the seed tissues (Figure 7). Non-transformed *Arabidopsis* Col-0 plants were used as a control to compare the GUS staining. Staining results showed dark blue color only in the pericarp (outer covering of siliques). In all other tissues, staining was not present except a minute staining in flowers in rare cases. These results clearly showed that *AhGLP17-1P* is abundantly expressed in the pericarp and almost no expression in other tissues. Overall, the results strongly suggest that this promoter is a suitable candidate to guide the expression of a gene in a pericarp-specific manner.

## Discussion

Constitutive expression of a gene in transgenic plants results in an additional metabolic burden on the plant system, and constitutive promoters can produce undesired phenotypes (Yuan et al., 2019b) and reduced production. Plants need to direct the valuable resources to the target areas for survival and smooth growth under normal and stressed conditions (Divya et al., 2019). Therefore, tissue-specific or stress-inducible promoters are ideal for altering the plants’ genetic architecture to perform better according to a researchers’ desired ideotype. Previous crop biofortification programs that resulted in present-day purple embryo maize (Liu et al., 2018), purple endosperm rice (Zhu et al., 2017), golden rice (Paine et al., 2005), and *Brassica juncea* for fish oil docosahexaenoic acid (DHA) production (Wu et al., 2005) were carried out to introduce new metabolic pathways in endosperm and seeds of these crop species by employing endosperm and seed-specific promoters. For example, a strong endosperm-specific rice glutenin *GluT01* promoter (*Glu*) was used to drive a novel rice phytoene synthase (*psy*) gene and *Erwinia uredovora crtI* gene fused with pea Rubisco small subunit plastid peptide to produce the high amount of β-carotene in Golden Rice (Paine et al., 2005). As peanut is an important oil and protein providing crop and primary source of nutrition in many Asian and African countries, it is prone to many biotic and abiotic stresses (Zhang et al., 2017). Changing its genetic makeup is key for its better performance under stressed conditions. The pericarp is a non-edible part of peanut seeds and serves as the first layer of defense against pathogens. Using pericarp-specific promoters to drive resistance-related genes is ideal for improving its fighting ability against soil and seed-borne pathogens and diseases. Although, in recent years, some studies have reported the identification and functional characterization of seed-specific promoters and genes in peanut (Yuan et al., 2019a; Yuan et al., 2019b; Tang et al., 2021), but no detail is viable for pericarp-specific promoters.

Therefore, the current study was based on identifying and functionally characterizing the promoter of a gene with unique expression in peanut pericarp and no/minimum expression in all other tissues. We identified a pericarp-specific gene (germin-like protein subfamily 1 member 7) by available microarray and transcriptome expression data. Germin-like proteins GLPs are a group of well-known proteins ubiquitously found in the plant kingdom. GLPs are “cupin superfamily” domain-containing proteins, composed of β-sheet barrel structure and metal ion binding site at their C-terminus (Dunwell et al., 2008; Agarwal et al., 2009). GLPs actively participate in plant defense against various fungal, bacterial, and viral pathogens (Godfrey et al., 2007; Knecht et al., 2010; Guevara-Olvera et al., 2012). We checked the expression specificity of the *AhGLP17-1* gene in different tissues by semiquantitative and qRT-PCR using a widely grown peanut cultivar, “Minhua-6”. Further, we cloned the promoter region of the *AhGLP17-1* gene from a high-yielding and disease-resistant cultivar, “Xinhuxiaoli”. We used two different peanut varieties as Minhua-6 is a largely cultivated variety, and our microarray expression is based on this cultivar. Its samples were easily available for RNA extraction and expression verification. At the same time, Xinhuixiaoli cultivar (disease-resistant; fungal and bacterial diseases), was used for cloning of promoter based on its possible future use to develop transgenic peanut for disease resistance. Online databases for promoter analysis predicted many *cis*-acting elements, including core promoter element as TATA Box and proximal control elements as CAAT Box, GC Box (Muthusamy et al., 2017), and many other light-responsive, hormones responsive, growth and regulation responsive, and stress-responsive elements. The number and types of *cis*-regulatory elements are important determinants of promoter strength and specificity (Stålberg et al., 1993; Tang et al., 2015). RY-repeat elements, known for seed-specific expression (Fujiwara and Beachy, 1994), were also present in the promoter region of the *AhGLP17-1* gene. One copy of (CA)n element “CNAACAC” was also found in the promoter region. (CA)n is known to be involved in the embryo and endosperm-specific transcription (Ellerström et al., 1996). But, to date, not a single element has been reported to be involved in the pericarp-specific expression. From *in silico* analysis of *AhGLP17-1P*, some unnamed elements were found that include Unnamed_1 (GAATTTAATTAA), Unnamed_2 (AACCTAACCT), Unnamed_4 (CTCC), and Unnamed_6 (taTAAATATct). There is a possibility that some of these elements or some other element have role in pericarp-specific activity.

Peanut is a suitable crop for genomic studies, but the main bottleneck is difficulties in peanut transformation, so *Arabidopsis* becomes an ideal alternative for functional studies of genes and promoters (Grace et al., 2004; Zavallo et al., 2010; Sunkara et al., 2014). Here, we verified the pericarp-abundant behavior of a germin-like protein family gene (*AhGLP17-1*) of peanut. Peanut is affected by several biotic and abiotic stresses, and the pericarp is the primary defense organ that protects peanut seeds from stresses and harsh conditions. Hence, altering the composition of this organ can result in new peanut cultivars with enhanced defense capabilities. If stress-related genes under the control of pericarp-specific promoters are successfully transformed into peanuts, it will be a milestone achievement in peanut breeding.

Pod rot is a complex disease associated with several *Pythium species,* deteriorates young pods and seeds (Yu et al., 2019). *Aspergillus flavus* is a serious threat to food safety which causes crop yield and quality deterioration by producing aflatoxins (Deng et al., 2018). Similarly, gray mold disease of peanut caused by *Botrytis cinerea* (Alam et al., 2019), web blight disease of groundnut caused by *Rhizoctonia solani* (Ganesan and Sekar, 2011), and a huge seed-borne fungal microflora attack peanut seeds and deteriorates yield and quality. Their first target is the pericarp or pod; after that, these pathogens invade edible seeds. Changing the genetic makeup of peanut pods is an ideal solution to avoid the damages of these pathogens. Chitinases, stilbene/resveratrol synthase, glucanases are well-known genes showing resistance to bacterial and fungal diseases (Medeiros et al., 2018; Vestergaard and Ingmer, 2019; Loc et al., 2020; Ueki et al., 2020). These genes can be derived by pericarp abundant/specific promoter to show high expression in pericarp tissues. In this way, the defensive ability of this tissue can be enhanced to protect the edible seeds.

Similarly, there are several other seed-borne diseases and pathogens that attack the growing peanut kernels. The pericarp is a potential barrier against these diseases and pathogens (Cobos et al., 2018). Transformation of these genes under the control of pod-specific promoters into high-yielding varieties can improve their disease resistance. Our results showed that *the AhGLP17-1* promoter showed expression specificity in pericarp tissues. Although there are some variations in expression patterns like in peanut, this gene also showed some expression in roots and no expression in flowers. But in transgenic Arabidopsis plants, the *GUS* gene under *AhGLP17-1P* showed some staining in flowers in some cases and no staining in roots. These variations are possibly attributed to diverse species. We are fully convinced that the *AhGLP17-1P* promoter investigated in this study could potentially drive the resistance-related genes in pericarp specific manner and alter the peanut pericarp genetic architecture to protect the edible seed from biotic stresses and environmental stresses.

## Conclusion

In this study, we cloned and functionally characterized a novel pericarp-specific promoter (*AhGLP17-1P*) of peanut for the first time. This specifically expressed gene was cloned based on the microarray and transcriptome expression data. Both semiquantitative and qRT-PCR confirmed its pericarp-specific and abundant expressions. The GUS staining and qPCR analysis of the *GUS* gene under *AhGLP17-1P* in different vegetative and reproductive tissues/organs of transgenic *Arabidopsis* plants clearly showed its expression in pericarp tissues and no expression in all other tissues including, roots, seedlings, stem, leaf, seeds, except minute expression in flowers in some cases. Our studied promoter can potentially improve disease/pathogen resistance in transgenic peanuts and other agronomically important crops by employing resistance-related genes.

## Data Availability

The original contributions presented in the study are included in the article/Supplementary Material, further inquiries can be directed to the corresponding author.
