# Supplementary material for: Cloning and Functional Characterization of a Pericarp Abundant Expression Promoter (AhGLP17-1P) From Peanut (Arachis hypogaea L.)
Source: Front Genet. 2022 Jan 20;12:821281. doi: 10.3389/fgene.2021.821281 (PMC8811503; doi:10.3389/fgene.2021.821281)
Supplement: Supplementary file 1 [file DataSheet1.ZIP › Supplementary Table 1.docx]

Supplementary Table 1. Primers used in the study

| **Semi-quantitative PCR** | |
| --- | --- |
| sq-AhActin-F | GGCAAAAAGGACTCACAGGTTC |
| Sq-AhActin-R | CCTTGATGTGTCAGGAGACCAA |
| sq-AhGLP17-1-F | AACGAACTAGCAGGACTCAACAC |
| sq-AhGLP17-1-R | GATAACACCTGGATTCTGACTGC |
| **qRT-PCR** | |
| AhActin-F | GAGGAGAATCAGAAGCAAGTC |
| AhActin-R | CATATACAGCATAGCGGCACTC |
| q-AhGLP17-1-F | GCGAGCGTTCCATGCAGAAG |
| q-AhGLP17-1-R | GGCTGGGATCATAGGCAGAG |
| **Promoter amplification** | |
| AhGLP17-1P-F | GAGTGTCTGCTATATTGATGGAGTCAAG |
| AhGLP17-1P-R | GTGGCTTTGCAATTTATCCACGCTGCAG |
| **Promoter Gateway cloning** | |
| gwGLP17-1P-F | GGGGACAAGTTTGTACAAAAAAGCAGGCTTCGAGTGTCTGCTATATTGATGGAGTCAAG |
| gwGLP17-1P-R | GGGGACCACTTTGTACAAGAAAGCTGGGTCGTGGCTTTGCAATTTATCCACGCTGCAG |
| **Verification of transgenic Arabidopsis plants** | |
| Pro-GUS-F | ATGCAAGCTAAGAGGCCAATTC |
| Pro-GUS-R | CGGCAATAACATACGGCGTGAC |
| **Quantitative expression of GUS gene** | |
| q-GUS-F | AGGATTCGATAACGTGCTGATGGTG |
| q-GUS-R | TCAATCACCACGATGCCATGTTCATC |
| AtActin-F | GGTAACATTGTGCTCAGTGGTGG |
| AtActin-R | GCAGCATGAAGATTAAGGTCGTT |
