# Supplementary material for: Cloning and Functional Characterization of a Pericarp Abundant Expression Promoter (AhGLP17-1P) From Peanut (Arachis hypogaea L.)
Source: Front Genet. 2022 Jan 20;12:821281. doi: 10.3389/fgene.2021.821281 (PMC8811503; doi:10.3389/fgene.2021.821281)
Supplement: Supplementary file 1 [file DataSheet1.ZIP › Supplementary Table 2.docx]

Supplementary Table 2. Microarray expression data of *AhGLP17-1* gene (AH06G08990.1) in different tissues.

| Gene/Probe ID | Root | Stem | Leaf | Flower | Gynophore | Pericarp-I | Pericarp-II | Pericarp-III | Testa | Embryo-I | Embryo-II | Embryo-III |
| --- | --- | --- | --- | --- | --- | --- | --- | --- | --- | --- | --- | --- |
| UN_p_total_uni_30450-1 | 27147.3 | 335.27 | 132.19 | 474.01 | 15436.5 | 27927.5 | 40624.2 | 28988.2 | 3306.49 | 62.9 | 18.78 | 20.64 |
| UN_p_total_uni_30450-2 | 634.65 | 243.78 | 96.53 | 151.92 | 1106.97 | 505.11 | 1753.18 | 2205.92 | 176.45 | 14.59 | 28.52 | 23.96 |
| UN_p_total_uni_30450-3 | 4394.91 | 4466.28 | 4403.22 | 4489.87 | 7536.25 | 11408.4 | 12695.6 | 12966.3 | 341.6 | 852.06 | 757.73 | 563.04 |

Note;

Pericarp-1= pericarp samples 10-20 days after pegging

Pericarp-II= pericarp samples 30-40 days after pegging

Pericarp-III= pericarp samples 50-60 days after pegging

Embryo-I= Embryo samples 20 days after pegging

Embryo-II= Embryo samples 30-40 days after pegging

Embryo-III= Embryo samples 50-60 days after pegging

Testa samples of 20 days, 30-40 days, and 50-60 days were mixed for microarray expression analysis.
