# Supplementary material for: Cloning and Functional Characterization of a Pericarp Abundant Expression Promoter (AhGLP17-1P) From Peanut (Arachis hypogaea L.)
Source: Front Genet. 2022 Jan 20;12:821281. doi: 10.3389/fgene.2021.821281 (PMC8811503; doi:10.3389/fgene.2021.821281)
Supplement: Supplementary file 1 [file DataSheet1.ZIP › Supplementary Table 3.docx]

| Tissue | FPKM |
| --- | --- |
| Leaf | 0.24 |
| Stem | 0.11 |
| Stem tip | 0.13 |
| Root | 65.72 |
| Root tip | 11.06 |
| Root nodule | 5.93 |
| Root and stem | 2.89 |
| Florescence | 0.46 |
| Gynophore | 11.03 |
| Pericarp-Ⅰ | 78.64 |
| Pericarp-Ⅱ | 1523.43 |
| Pericarp-Ⅲ | 1076.32 |
| Testa -Ⅰ | 0.49 |
| Testa -Ⅱ | 0.45 |
| Embryo-Ⅰ | 0.31 |
| Embryo -Ⅱ | 0.35 |
| Embryo -Ⅲ | 0 |
| Embryo-Ⅳ | 0 |
| Cotyledon | 0.33 |

Supplementary Table 3. Transcriptome expression data of *AhGLP17-1* gene (AH06G08990.1) in different tissues.

Note;

Pericarp-1= pericarp samples 10-20 days after pegging

Pericarp-II= pericarp samples 30-40 days after pegging

Pericarp-III= pericarp samples 50-60 days after pegging

Embryo-I= Embryo samples 10-20 days after pegging

Embryo-II= Embryo samples 20-30 days after pegging

Embryo-III= Embryo samples 30-40 days after pegging

Embryo-IV= Embryo samples 50-60 days after pegging

Testa-I= Testa samples 30 days after pegging

Testa-II= Testa samples 50 days after pegging
