# Supplementary material for: Cloning and Functional Characterization of a Pericarp Abundant Expression Promoter (AhGLP17-1P) From Peanut (Arachis hypogaea L.)
Source: Front Genet. 2022 Jan 20;12:821281. doi: 10.3389/fgene.2021.821281 (PMC8811503; doi:10.3389/fgene.2021.821281)
Supplement: Supplementary file 1 [file DataSheet1.ZIP › Supplementary Table 4.docx]

Supplementary Table 4. Functional Annotation of *AhGLP17-1* Gene

| GO category | GO ID | GO term |
| --- | --- | --- |
| Biological process | GO:0033609 [Go To GO] | oxalate metabolic process |
| Molecular function | GO:0045735 [Go To GO] | nutrient reservoir activity |
|  | GO:0046564 [Go To GO] | oxalate decarboxylase activity |
|  | GO:0030145 [Go To GO] | manganese ion binding |
| Cellular component | GO:0048046 [Go To GO] | apoplast |
|  | GO:0005618 [Go To GO] | cell wall |
|  | GO:0005576 [Go To GO] | extracellular region |
