# Supplementary material for: Cloning and Functional Characterization of a Pericarp Abundant Expression Promoter (AhGLP17-1P) From Peanut (Arachis hypogaea L.)
Source: Front Genet. 2022 Jan 20;12:821281. doi: 10.3389/fgene.2021.821281 (PMC8811503; doi:10.3389/fgene.2021.821281)
Supplement: Supplementary file 1 [file DataSheet1.ZIP › Supplementary Table 5.docx]

Supplementary Table S4. Additional information of *AhGLP17-1* gene

| 1st NR ID | gi\|147787462\|emb\|CAN71140.1\| |
| --- | --- |
| 1st NR Description | hypothetical protein VITISV_005471 |
| 1st NR Species | *Vitis vinifera* |
| 2st NR ID | gi\|15230587\|ref\|NP_187246.1\| |
| 2st NR Description | germin-like protein subfamily 1 member 7 |
| 2st NR Species | *Arabidopsis thaliana* |
| A duranensis | Aradu.K66PA |
| A ipaensis | Araip.B0Q1D |
| Arabidopsis thaliana | AT3G05950.1 |
| Glycine max | GLYMA16G06500\|GLYMA16G06500.1 |
| Oryza sativa | LOC_Os08g09040.1 |
