# Supplementary material for: Cloning and Functional Characterization of a Pericarp Abundant Expression Promoter (AhGLP17-1P) From Peanut (Arachis hypogaea L.)
Source: Front Genet. 2022 Jan 20;12:821281. doi: 10.3389/fgene.2021.821281 (PMC8811503; doi:10.3389/fgene.2021.821281)
Supplement: Supplementary file 1 [file DataSheet1.ZIP › Suppelemtary file 1.docx]

**Supplementary file 1. CDS, Protein, and promoter sequences of AhGLP17-1 gene.**

**Peanut Gene ID** AH06G08990

**Peanut Transcript ID** AH06G08990.1

**Description** Germin-like protein subfamily 1 member 7

Sequences

**CDS**

>AH06G08990.1

ATGCAGAAGATATTCAAGTCTCTCACAATTAACAAGCAATTATATAGACAGCATCATACAAAACTAAAAATGAAAGCTGCATACTTGCTGGTTGCATTCTTGGCTCTGGCCTCTTTTGCCTCTGCCTATGATCCCAGCCCTCTCCAAGACTTTTGTGTTGCTCTCCCCGATGGCAGCATCAAAGACGCTGTATTTGTGAACGGAAAATTTTGCAAAGACCCTAAAATTGTGGTAGCTGAGGATTTTTTCAAGCACGTAGATCCTGGGAATGTTGTTAACAAACTTGGATCAAAAGTAACTCCAGTGACAGTTAACGAACTAGCAGGACTCAACACATTGGGTATATCACTTGCTCGCATAGATTTTGGATCTAAGGGTTTAAATCCTCCTCACACTCACCCTCGAGGCACTGAGGTATTGATAGTTATTGAAGGAACTCTCTTAGTTGGATTTGTGACTTCCAATCAGAACAACACCAACCGTCTTTTTACCAAATGCTCAACAAGGGTGATGTGTTTGTGTTCCCAATTGGTCTCATTCATTTCCAATTCAACGTCGGTTATGGCAACGCTGTTGCTATTTCTGGACTTAGCAGTCAGAATCCAGGTGTTATCACAATTGCAAATGCTATTTTTGGATCCACTCCACCTATTTCTCCTGAAGTTTTGA

**Protein**

>AH06G08990.1

MQKIFKSLTINKQLYRQHHTKLKMKAAYLLVAFLALASFASAYDPSPLQDFCVALPDGSIKDAVFVNGKFCKDPKIVVAEDFFKHVDPGNVVNKLGSKVTPVTVNELAGLNTLGISLARIDFGSKGLNPPHTHPRGTEVLIVIEGTLLVGFVTSNQNNTNRLFTKCSTRVMCLCSQLVSFISNSTSVMATLLLFLDLAVRIQVLSQLQMLFLDPLHLFLLKF

**Promoter**

>AH06G08990.1

GAGTGTCTGCTATATTGATGGAGTCAAGATAGATCCATGCCACCAAGCACCATCTAGGCTGAACAAGTTTTTTTATTTTATTTTTTTAGAAGAGGTTTTCAATCATCGTTTGTTGCACAATATACATCACTGAACTGGAGACGCTGATCAACATAACCTCGCCACCATCGAGAAAAAAAAGAAAAATAAATAGTGGGATATATGTGTATATAATAAGTTGGATTAAAGATAAGGATAAATAGTGAGATTCGCACAACAACAAAGAGACAGTAGATGGTAAGCTAACTCATATTATTAAAGTGAATCATCAGAAATAGATATAGGAAATTAGGTTTAAATGGGAACATCAGAAAAGAATCACAATCCCTCCATTGGATATTCCAGGCTGCTGTGCTTAAATTTATATATTTGCTTCATGAAACATACTTTGGAGTCAACTACTGTACTCTCAAGGAAAATTTTAAAGAAAAATATAGGTAAACAATAAAAATATTAAACAATATAAACAATAGACATATCAGATATTTAATTCACTAGTTGTGCATATGGTTATTTTAATATTAAGATTTAGATGAGTAATTTGGAGATATAATGTGTTTTATTTTCAATTTTAGAGCTCGTTGTTCATGTTATTCAAAAAAGTCATTAATTATTTAATATAATCCAAATTTTAATTCTTTACCATATAACAACTCTCTATTTTTTTCATTATAACTAACCCTACCTTACATATTCATTTGGATTTCTTCAAATTTAATTAACTTCCGCTACAATATAATAAATCATTTAAAGGACACCAAACAATTACTAACGAACTTTCCTTGTGAGTTGTGACTATAAATTAGTGGAGGTCAGTGTTTGACCCATATAATTGCTTACAACTTTTTCCCTTTAACCCTAACTGCTCTTGACATTTTGTAGACACACAAGCTAACTTTCTATATAAAGATCGCACTATGTATGGTCAAATGGATAAGCCTAGAAGAGGCCCAATTACTTTAAAGAATAATGTAATTTACTAATTCATTAGTTCTAAAAGGACACATCAAATTTTAGCGACTAGGTCTCAAGAATATATACAAACTCCGAAGCTATTCTCAACTTCAGTTTCCTAGTTGCCTTTAAGAAATTAACTAATTGCTTATGTATTTTTTGTTAAAATTATACAAAATAATCATATATTTAACTTATTCGATATATAGTTAAAGTTTAATAAAAATAAGATTTTGAAATATGCATTAAATATACTCTAAAACCACTGAATAATATTTCATAGTCTTGTGAGAATAAAATAATATAATAAATTAATTATTTAGGATTTAGTTTACATAATACTCTATATTTAAATAAAGAAAAAGTCTAGAAGATCAACTAAAGTGTAACCAACTAAGAGTCAATTAATTAAATTTTTTTATTTTTAATTTTAAAATTTTAAAAATTATGATAGTGAGAGTTGTTTTTTTTGTTGTAATAAACTTATTATCTAACTAGTTGGCTCTAAGTTAAATATTTCAAATTGGTTTCTTAGCAGGATCGATAAATAAAATATACAGAGATATTTATTTCTTATGTTAAATACATACACAATTAATTTATTATTCAATAGTCACCAAAAAAAATTATTATTCAATAATATATAAAAATATTTTTGACTTTTACTTTATTCAATGCATTAAACTACGTTTGGTTTTAAAAAGGGTCGAATTATAATTAATTAATCAATTACATAGATAATTATTATTGAAACTTTTTATCAAATTAAATTGATTATATAAACTTAACCCTTTTCTCATTTATTTATTTGAGTATTTATTGTCTCTCTACCTACATATTATTAGTACCAATTAATTAACAACTAATAATTAAAATAAAGATTTTATTTTAATTAAAATTAAATTGATTATATAAACTTACCCATTTTTCTTATTTATTTATATGAGTATTTACTCTCTCTACCTATGTATTATATTAGTACCAATTAATTAACAAAAAGTTTGTTAAATGTTACCTAATTTTTTCGAAAAAAAATTGACCATCAAATAACACTTTTAAAGTGAGTACTTATTTTAAATCTGTAGATAAAATGAATGCAAGCTAAGAGGCCAATTCAACGTGAGATCAGATTATATACCAAAAATAATTCCATTGGCTTTAACTTATGGGAAGCATCTTCTTATATTTTAAAATATATTCTTTGAACTAGCATGCACGTAGTTGTTGAAGAATGGTTCCACAATGCAAATACTATAGAATAATATTAATTACCTACTGCAGCGTGGATAAATTGCAAAGCCACTTCAATGGTTCCATGCTATAAATAGCGAGCGTTCC**ATG**

**Note:** in promoter sequence, underlined region is 5’ UTR while yellow highlighted regions are forward and reverse primers.
